# Fibrous Dysplasia Developed at the Site of a Previous Central Giant Cell Granuloma in a Middle‐Aged Patient: A Case Report

**DOI:** 10.1155/crid/3449150

**Published:** 2026-07-18

**Authors:** Mahdi Niknami, Nazanin Mahdavi, Zahra Madani

**Affiliations:** ^1^ Department of Oral & Maxillofacial Radiology, School of Dentistry, Tehran University of Medical Sciences, Tehran, Iran, tums.ac.ir; ^2^ Oral and Maxillofacial Pathology Department, School of Dentistry, Tehran University of Medical Sciences, Tehran, Iran, tums.ac.ir; ^3^ Department of Oral & Maxillofacial Radiology, School of Dentistry, Arak University of Medical Sciences, Arak, Iran, arakmu.ac.ir

**Keywords:** case report, fibrous dysplasia, giant cell, granuloma, middle-aged

## Abstract

**Background and Aim:**

Fibrous dysplasia (FD) is a benign fibro‐osseous condition, characterized by replacement of normal bone with immature fibrous tissue due to GNAS mutations, whereas central giant cell granuloma (CGCG) is a reactive lesion featuring multinucleated giant cells in a vascular stroma. Although some hybrid lesions combining FD and CGCG have been reported as synchronous entities, metachronous occurrences, where FD develops years after CGCG excision, are rare. This report describes a case of metachronous FD arising at the site of a previously excised CGCG after a 10‐year interval, exploring potential temporal association and emphasizing the need for extended follow‐up.

**Case Presentation:**

A 50‐year‐old woman presented with a firm, gradually enlarging swelling in the right maxilla that had developed over 2 years. Her history included surgical excision of a pure CGCG (no fibro‐osseous elements) in the same location 10 years prior. Clinical examination revealed bony‐hard expansion without neurosensory deficits or lymphadenopathy. Cone‐beam computed tomography showed a poorly defined, expansile radiopaque mass with a ground‐glass pattern, obliterating the maxillary sinus. An incisional biopsy showed evidence of FD, with irregular woven bone trabeculae in fibrous stroma lacking osteoblastic rimming.

**Conclusion:**

To the best of our knowledge, this is one of the first reported metachronous cases of FD arising de novo at the site of a previously excised CGCG following a 10‐year interval. This report underscores the importance of long‐term follow‐up for patients with jaw pathologies to detect rare sequential developments.

## 1. Introduction

Fibrous dysplasia (FD) is a noninherited genetic condition in which normal bone is progressively replaced by immature and irregularly arranged fibro‐osseous tissue, often leading to deformities, pain, fractures, and impaired function [1]. The pathology arises from a sporadic postzygotic mutation in the *GNAS* gene, which encodes the *α*‐subunit of the Gs stimulatory protein. This mutation results in structurally weak fibrous tissue distorting the affected bone [2]. FD represents approximately 5%–7% of benign bone tumors. It is classified as monostotic, involving a single bone, or polyostotic, affecting multiple bones. The craniofacial skeleton is frequently involved, with the maxilla and zygomaticomaxillary region being the most commonly affected sites in the jaws [1, 2].

Mild, asymptomatic FD is often detected incidentally on imaging, and lesions are typically poorly defined, complicating diagnosis. While monostotic FD is common, comprehensive evaluations are not always performed, making it challenging to assess true prevalence [3, 4]. FD can present at any age, but it is typically diagnosed in adolescents and young adults. A recent systematic review on craniofacial FD reported a mean patient age of 21.6 years at the time of assessment [5].

Central giant cell granuloma (CGCG) predominantly occurs in younger individuals and shows a higher incidence in females. It is most frequently located in the mandible and tends to affect the anterior region more often than the posterior jaws. It presents as an asymptomatic expansion and has a variable radiographic appearance [6, 7]. CGCG is largely considered a reactive or reparative process rather than a true neoplasm. Its clinical behavior ranges from indolent growth to more aggressive forms that cause pain and root resorption [8].

Hybrid lesions are rare entities characterized by the presence of two or more distinct pathologies within a single lesion. Conventionally, this term implies that both components occur simultaneously or synchronously. The literature has reported cases in which CGCG coexists with fibro‐osseous lesions, including FD, cemento‐osseous fibroma, cemento‐osseous dysplasia, and Paget′s disease [9–12]. However, the existing literature does not address the possibility of a sequential relationship, where one lesion develops years after the other at the same anatomical site. This creates a gap in our understanding, raising questions about the long‐term pathogenic potential of jaw tissues following the treatment of a primary lesion. The development of FD at the precise location of a surgically treated CGCG after a decade‐long disease‐free interval challenges the notion that these pathologies must occur together to be related. Therefore, this report is aimed at presenting a case of FD that developed at the site of a previously excised CGCG after a 10‐year interval. We discuss the diagnostic challenge and explore the potential pathogenic relationship between these two entities, highlighting the clinical importance of this unprecedented temporal separation.

## 2. Case Presentation

### 2.1. Patient Presentation and History

A 50‐year‐old woman was referred to the Department of Oral and Maxillofacial Pathology at Tehran University of Medical Sciences for evaluation of a slowly progressive swelling in the right maxilla. The patient reported that the swelling had been present for approximately 2 years. The patient reported a history of previous surgery in the same area for a different pathology 10 years earlier. The pathology report from that previous surgery indicated that the lesion was a CGCG, with no evidence of FD, and an excisional biopsy had been performed. The patient′s medical history was otherwise unremarkable. She denied any history of trauma to the maxillofacial region, prior head and neck radiation therapy, or other systemic diseases, including recent significant illnesses such as COVID‐19. She was postmenopausal and not taking hormone replacement therapy. Ethical approval was waived by the institutional review board of Tehran University of Medical Sciences. Written informed consent was obtained from the patient for publication of this case report and accompanying images.

### 2.2. Clinical and Radiographic Findings

On extraoral examination, a notable, bony‐hard swelling was palpable in the right malar region. However, there were no indications of neurosensory deficits or lymphadenopathy. Intraoral examination revealed a firm, nontender, bony enlargement with normal‐colored overlying mucosa. The lesion extended from the midline of the maxilla to the tuberosity region, expanding both the buccal and palatal cortical plates. The patient reported some interference with occlusion and mastication due to the expansion.

Panoramic imaging revealed an ill‐defined radiopaque region in the right maxilla, exhibiting heterogeneous opacification and a characteristic ground‐glass appearance. There was no evidence of root resorption, periodontal ligament widening, or periosteal reaction. To further characterize the lesion, a cone‐beam computed tomography (CBCT) scan was performed. The CBCT showed the presence of an ill‐defined, expansile mass that obliterated the right maxillary sinus and caused a slight buccal displacement of the maxillary right incisor (Figure 1).

**Figure 1:**
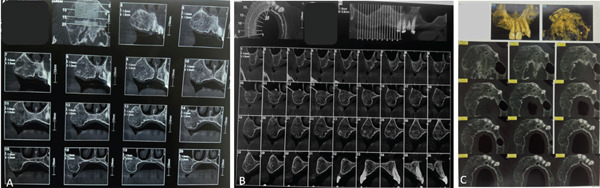
Cone‐beam computed tomography (CBCT) images of the right maxilla. (A) The coronal view demonstrates the expansion of the lesion into the right maxillary sinus, elevating its floor. (B) The cross‐sectional view shows the significant buccal and palatal expansion of the alveolar process. (C) The axial view displays the ill‐defined borders of the lesion and its characteristic ground‐glass radiopacity, which has replaced the normal trabecular bone pattern.

### 2.3. Differential Diagnoses

Based on the patient′s age, the clinical features of a slow‐growing maxillary expansion, and the ground‐glass radiographic appearance, a list of differential diagnoses was formulated. This included FD, a recurrent CGCG with osseous changes, a cemento‐ossifying fibroma, chondrosarcoma, and a low‐grade osteosarcoma.

### 2.4. Surgical Intervention and Biopsy

Following written informed consent, an incisional biopsy was performed under local anesthesia using 2% lidocaine with 1:100,000 epinephrine, administered via infiltration into the vestibular mucosa and hard palate of the right maxilla. After obtaining written informed consent, incisional biopsy was carried out under local anesthesia was achieved using 2% lidocaine with 1:100,000 epinephrine, which was injected into the right maxillary buccal vestibule and the hard palate. Access to the right maxillary alveolar bone was achieved through flap elevation. Using osteotomes, some areas of bony expansion were removed. After ensuring hemostasis in the surgical field, the area was irrigated with copious amounts of saline. The intraoral incision was then sutured with 4‐0 Vicryl.

To investigate the potential relationship between the current and previous lesions, the original slides from the surgery 10 years prior were retrieved and re‐examined. The diagnosis was established based on the characteristic microscopic features observed in H&E‐stained sections and supported by CD68 immunohistochemical positivity. This retrospective analysis showed the diagnosis of CGCG, characterized by a proliferation of mononuclear stromal cells and numerous multinucleated giant cells scattered throughout the tissue at low (40×) and high (200×) magnification (Figure 2). Critically, there was no evidence of FD or any other fibro‐osseous component in the original lesion. While independent expert pathological review supported the diagnosis, the interpretation remains based on routine histopathological assessment, and additional molecular characterization was not available.

**Figure 2:**
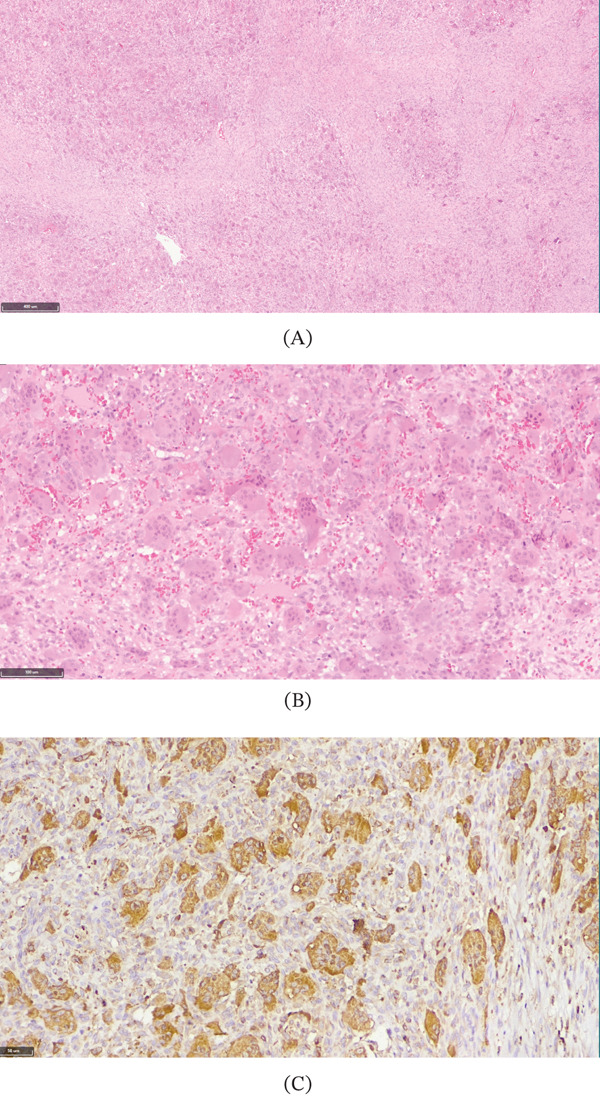
(A) Low‐power photomicrograph of central giant cell granuloma demonstrating the overall architecture of the lesion with a cellular fibrovascular stroma and variably distributed giant cell‐rich areas (H&E, ×40). (B) Photomicrograph reveals proliferation of multinucleated giant cells within a cellular mesenchymal stroma with prominent capillaries and RBCs extravasation (H&E, ×200) (C) Cytoplasmic expression of CD68 in multinucleated giant cells in CGCG is notable (×200).

The specimen received for examination consisted of multiple light brown, hard tissue fragments, with an aggregate size of approximately 1.4 × 0.5 × 1.0 cm. The microscopic examination revealed irregular trabeculae of woven and lamellar bone embedded in a moderately cellular fibrous stroma. These trabeculae displayed the classic curvilinear, “Chinese letter” pattern and lacked prominent osteoblastic rimming (Figure 3). These findings were compatible with FD; however, the fibrocellular stroma was limited, and the interface with native bone was not fully represented in the available specimen. Therefore, the diagnosis should be interpreted as the most likely clinicopathologic diagnosis rather than as unequivocal histopathological proof. Additionally, abundant sheets of mature adipose tissue were evident among the bone trabeculae, indicating fatty metamorphosis (Figure 3). Overall, the histopathological findings were considered most consistent with FD after integration of the clinical and radiographic findings, and the diagnosis was independently supported by two oral pathologists. However, the absence of a clearly demonstrable interface between lesional and native bone represents an acknowledged limitation of the available biopsy specimen. Masson′s trichrome staining was additionally performed on the histological sections, and the corresponding microscopic images are shown.

**Figure 3:**
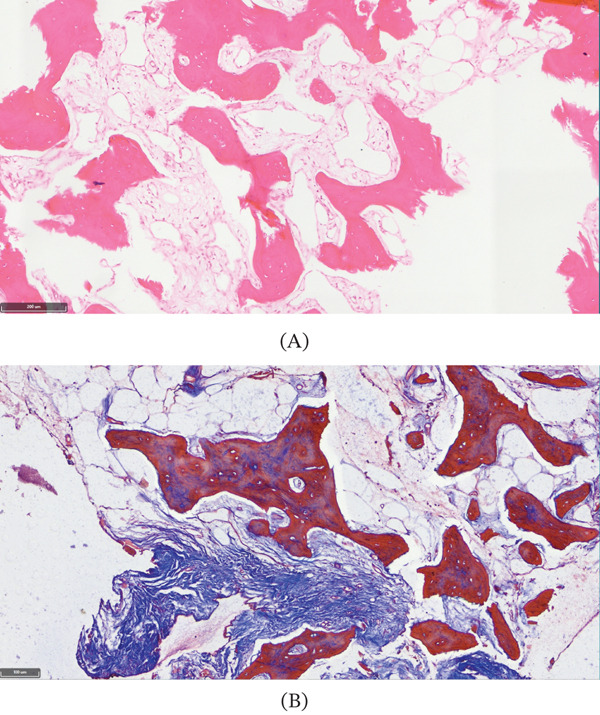
(A) The microscopic examination revealed irregular trabeculae of woven and few lamellar bone within in a loose fibro‐fatty stroma. (B) Masson trichrome stain showing irregular curvilinear bony trabeculae (red) surrounded by collagen‐rich fibrous stroma (blue), highlighting the characteristic fibro‐osseous architecture of fibrous dysplasia (×100).

### 2.5. Treatment and Follow‐Up

Given the monostotic nature and asymptomatic presentation postbiopsy, the patient was managed conservatively with regular clinical and radiographic monitoring. No further surgical intervention was required. At 2‐year follow‐up, the lesion showed no signs of recurrence, progression, or malignant transformation, and the patient reported no symptoms.

## 3. Discussion

The remarkable finding of this case is the development of FD at the exact anatomical site of a CGCG that had been surgically excised 10 years prior. This sequential presentation is a departure from the conventional understanding of hybrid lesions, which are usually defined by the synchronous occurrence of two distinct pathologies within the same mass. A definitive diagnosis of such complex cases requires careful integration of the patient′s history in conjunction with clinical, radiographic, and histopathologic findings.

Hybrid odontogenic lesions are rare. The majority of hybrid lesions arise in the mandible, predominantly in the posterior region, and are more commonly observed in females [9]. The case described here is rare in terms of its location and the age of the patient, as well as the occurrence of the lesions with a 10‐year interval. In our report, the patient, a 50‐year‐old female, presented with a lesion in the posterior maxilla, highlighting an unusual temporal and anatomic association between two distinct lesions. The first theory describes a “collision tumor,” in which both lesions occur simultaneously. The second theory proposes that a primary neoplasm develops initially, and the subsequent release of growth factors and chemokines by this lesion leads to the formation of a metachronous occurrence. The third theory suggests that following the development of the primary lesion, external stimuli, such as tissue trauma, trigger the emergence of a second lesion [13]. Although these hypotheses have been proposed to explain synchronous hybrid lesions, their applicability to the present metachronous case remains uncertain. The temporal sequence observed in our patient does not establish a causal relationship between the previous CGCG and the subsequent FD. Rather, this case demonstrates only a temporal association between two histopathologically distinct lesions occurring at the same anatomical site. Whether this represents a true biological association or two independent pathological events cannot be determined from the available clinical and histopathological evidence. No direct evidence supporting these mechanisms was available in the present case. Furthermore, molecular investigations were not performed, precluding assessment of any shared pathogenic pathway. Because molecular analyses were not performed, it was not possible to determine whether the two lesions shared any underlying biological relationship or whether their occurrence at the same anatomical site represented independent events. As highlighted in a recent review by Aliu et al., CGCG is a therapeutic challenge known for its high recurrence rates, often necessitating thorough surgical intervention to prevent its return [14]. Any systemic or host‐related contributors remain speculative and cannot be inferred from this report [15].

Histologically, FD is characterized by irregularly shaped bony trabeculae that are not interconnected, showing a characteristic “Chinese letter” pattern. These trabeculae lack osteoblastic rimming and are embedded within a fibrous stroma, often accompanied by extravasated erythrocytes and scattered giant cells. Fusion of the lesional tissue with the surrounding uninvolved bone is a hallmark feature of FD. The presence of multinucleated giant cells, seen in both FD and CGCG, may provide insight into their nature. Although the exact etiology of CGCG in the jaw remains unclear, it has been proposed that it may arise from an exaggerated reparative response to previous trauma and intraosseous hemorrhage, leading to a reactive granulomatous process [10]. In hybrid lesions involving giant cell tumors associated with FD or other fibro‐osseous lesions, it has been proposed that the giant cells may react to stromal alterations within the original tumor. These changes in the stroma could stimulate osteoblasts, which may subsequently activate osteoclast‐like giant cells via a paracrine mechanism [9, 16].

Hybrid CGCG lesions associated with FD differ from “nonhybrid” FD in that they more commonly arise in the posterior mandible and often exhibit a multilocular radiolucent component. According to a systematic review, “nonhybrid” FD typically presents unilaterally in the maxilla and appears radiographically as a diffuse radiopacity, with 52% of cases showing radiopaque features compared to only 5% displaying radiolucency [17]. As mentioned, our case showed “nonhybrid” FD more than a hybrid lesion, as it was an expansile opaque lesion, and the radiological characteristics typical of hybrid lesions, such as radiolucent and multilocular features, were not present. In all reported cases of hybrid lesions exhibiting features of both CGCG and FD, the lesions occurred concurrently (Table 1), with examples including Kurra et al. [10]. Our case represents a rare sequential (metachronous) occurrence distinct from these synchronous hybrids.

**Table 1:** Demographic data on the hybrid‐central giant cell granuloma lesions.

Author, year	Hybrid lesion	Age (years old)	Sex	Jaw	Treatment and follow‐up
Yáñez et al. 2021 [18]	Monostotic FD	47	Female	Right mandible (body and ramus)	Incisional biopsy + likely conservative management
Jawanda et al. 2015 [19]	FD	33	Male	Mandible	Excisional biopsy
Kurra et al. 2013 [10]	FD	18	Female	Mandible	Surgical removal
Farzaneh et al. 2005 [16]	FD	20	Female	Mandible	Biopsy → lesion removal
Rahimov et al. 2013 [20]	FD	12	Male	Mandible	NA

*Note:* Prior reports describe synchronous hybrids; our case is metachronous.

Abbreviations: FD, fibrous dysplasia; NA, not available.

A major limitation of this report is that it describes a single observational case, which precludes any inference regarding a causal or biological relationship between the previously excised CGCG and the subsequently diagnosed FD. The present findings support only a temporal association between the two lesions. Additionally, the absence of molecular studies, such as GNAS sequencing, limits insights into potential shared genetic drivers. However, it introduces a previously undocumented clinical scenario and highlights a significant gap in our understanding of the long‐term sequelae of jaw pathologies. Moreover, while no definitive immunohistochemical markers distinguish FD from CGCG, the diagnosis of FD was established through integration of the clinical, radiographic, and histopathological findings rather than histopathology alone. Although the biopsy demonstrated characteristic curvilinear trabeculae lacking osteoblastic rimming, the classical interface between lesional and native bone was not represented in the specimen. Therefore, this feature should be interpreted as a limitation of the pathological assessment rather than evidence against the diagnosis. The final diagnosis was reached by consensus between two experienced oral pathologists after consideration of all available findings. Another important limitation is that no laboratory investigations, including serum calcium, phosphate, alkaline phosphatase, and parathyroid hormone measurements, were performed to exclude hyperparathyroidism or other metabolic bone disorders that may present with giant cell‐rich lesions of the jaws. Given the patient′s previous diagnosis of CGCG, the absence of this biochemical assessment reduces the completeness of the diagnostic workup and should be considered when interpreting the findings. Nevertheless, the clinical presentation, radiographic characteristics, independent review of the original CGCG specimen, and the histopathological features of the current lesion were considered most consistent with the diagnoses reported in this case. The potential mechanisms discussed in this report should be regarded as speculative hypotheses intended to generate future research questions. Given the observational nature of a single case report and the absence of molecular, biochemical, or experimental evidence, no causal relationship between the previous CGCG, surgical treatment, and subsequent development of FD can be established. Future perspectives should include greater awareness among clinicians and pathologists of this potential long‐term sequence. Reporting of similar cases is essential to determine if this is a recurring phenomenon and to better elucidate the complex interplay between local trauma, cellular signaling, and systemic factors like hormonal status in the pathogenesis of fibro‐osseous lesions, potentially through prospective studies with genetic profiling.

## 4. Conclusions

This report describes a case in which FD developed at the site of a previously excised CGCG, occurring 10 years after the initial surgery. Unlike previously reported synchronous hybrid CGCG‐FD lesions, this case documents a metachronous temporal sequence. However, the available clinical, radiographic, and histopathological findings support only a temporal association between these lesions and do not establish a causal or biological relationship. Accordingly, this case should be interpreted primarily as a clinicopathological observation that highlights a rare temporal association rather than as evidence of a causal or pathogenetic relationship between CGCG and FD. This case highlights the importance of long‐term surveillance following the treatment of benign jaw lesions, as the surgical site may remain a locus of future pathologic activity, and underscores that the interrelationship between these distinct pathologies is more complex than previously understood. Future research should focus on molecular analyses, such as GNAS mutation profiling in similar cases, to clarify shared pathways and inform targeted therapies, potentially reducing recurrence risks in high‐risk patients.

## Author Contributions

All coauthors have read and approved the final version of the manuscript.

## Funding

No funding was received for this manuscript.

## Consent

The authors certify that they have obtained all appropriate patient consent forms. In the form, the patient consented to his images and other clinical information being reported in the journal. The patient understands that his name and initials will not be published and due efforts will be made to conceal his identity, but anonymity cannot be guaranteed.

## Conflicts of Interest

The authors declare no conflicts of interest.

## Data Availability

The authors confirm that the data supporting the findings of this study are available within the article.
